# The PTEN and ATM axis controls the G1/S cell cycle checkpoint and tumorigenesis in HER2-positive breast cancer

**DOI:** 10.1038/s41418-021-00799-8

**Published:** 2021-05-31

**Authors:** Christian Bassi, Jerome Fortin, Bryan E. Snow, Andrew Wakeham, Jason Ho, Jillian Haight, Annick You-Ten, Emily Cianci, Luke Buckler, Chiara Gorrini, Vuk Stambolic, Tak W. Mak

**Affiliations:** 1 Princess Margaret Cancer Centre, University Health Network, 610 University Ave, Toronto, ON M5G 2C1 Canada; 2 Department of Pathology, Centre of Oncology and Immunology, The University of Hong Kong, Hong Kong, China; 3 Present Address: Epigenetics and Genome Stability Team, The Institute of Cancer Research, 237 Fulham Road, London, SW3 6JB UK; 4 Present Address: Paediatric Solid Tumour Biology and Therapeutics Team, The Institute of Cancer Research, 15 Cotswold Road, Sutton, London, SM2 5NG UK

**Keywords:** Tumour-suppressor proteins, Molecular biology

## Abstract

The tumor suppressor PTEN is disrupted in a large proportion of cancers, including in HER2-positive breast cancer, where its loss is associated with resistance to therapy. Upon genotoxic stress, ataxia telangiectasia mutated (ATM) is activated and phosphorylates PTEN on residue 398. To elucidate the physiological role of this molecular event, we generated and analyzed knock-in mice expressing a mutant form of PTEN that cannot be phosphorylated by ATM (PTEN-398A). This mutation accelerated tumorigenesis in a model of HER2-positive breast cancer. Mammary tumors in bi-transgenic mice carrying *MMTV-neu* and *Pten*^*398A*^ were characterized by DNA damage accumulation but reduced apoptosis. Mechanistically, phosphorylation of PTEN at position 398 is essential for the proper activation of the S phase checkpoint controlled by the PI3K–p27^Kip1^–CDK2 axis. Moreover, we linked these defects to the impaired ability of the PTEN-398A protein to relocalize to the plasma membrane in response to genotoxic stress. Altogether, our results uncover a novel role for ATM-dependent PTEN phosphorylation in the control of genomic stability, cell cycle progression, and tumorigenesis.

## Introduction

PTEN contains an N-terminal phosphatase domain that can dephosphorylate a component of the lipid cellular membrane, phosphatidylinositol 3,4,5-trisphosphate (PI(3,4,5)P_3_ or PIP_3_) [1]. By dephosphorylating the D3 position of PIP_3_, PTEN antagonizes the phosphatidylinositide 3-kinase (PI3K) pathway [1, 2]. The PI3K pathway regulates diverse cellular processes, including cell metabolism, survival, proliferation, apoptosis, growth, and migration. These fundamental cellular processes, when deregulated, can contribute or drive a malignant phenotype.

Somatic loss of function mutations of *PTEN* are found in a variety of human cancers including breast, endometrial carcinoma, glioblastoma multiforme, skin, and prostate cancers [3]. PTEN loss is a frequent event in breast cancer and is closely associated with accelerated progression and poor prognosis [4]. In particular, the expression of PTEN has been proposed to play an important role in human epidermal growth factor receptor 2 (HER2)-overexpressing breast cancers [5]. HER2 is a member of the epidermal growth factor receptor family which possess tyrosine kinase activity [6]. Its overexpression, observed in approximately 15–20% of breast cancer cases [7], is correlated with aggressive clinical behavior and poor prognosis [8]. Trastuzumab, a monoclonal antibody that binds with high affinity to the extracellular domain of HER2, is an effective therapy in HER2-positive breast cancer patients. The overall response rate reaches approximately 70% when PTEN expression is detected, but only about 20% for patients with negative PTEN expression [9, 10].

PTEN loss of function is often associated with genomic instability [11, 12]. Moreover, genetic deletion of *PTEN* in mouse embryonic fibroblasts (MEFs) causes accumulation of unrepaired DNA double-strand breaks [13]. PTEN loss is thought to contribute to genome integrity via at least two molecular mechanisms [14, 15]. In the nucleus, PTEN associates with the centromeric binding protein CENP-C and promotes kinetochore assembly and the metaphase-to-anaphase transition [13]. Further, acting as a co-factor for the transcription factor E2F1, nuclear PTEN appears to regulate the expression of Rad51, a key component of the DNA repair machinery [13]. However, follow-up work [16] has yielded inconsistent results, suggesting that PTEN regulation of RAD51 at the transcriptional level might be restricted to specific cell-contexts.

PTEN deficiency alter multiple cell cycle checkpoints, possibly leaving less time for DNA damage repair and/or chromosome segregation [16]. Progression through the cell cycle requires the flawless execution of several molecular processes performed in a timely manner to ensure a proficient, and error-free, cell division. The pace at which these events occur is dictated by the activity of cyclin-dependent kinases (CDKs), which phosphorylate key substrates to promote DNA synthesis and mitotic progression. The catalytic activity of CDKs is regulated by cell cycle checkpoints that monitor the orderly execution of the major events of the cell cycle. Checkpoints represent fail-safe mechanisms which ensure that cell division is licensed only when optimal circumstances are met [17]. Proper genome maintenance through cell division cycles is required for all organisms to ensure normal reproduction, development, and prevention of diverse diseases including cancer. DNA damage can arise from endogenous processes such as DNA mismatches that are occasionally introduced during DNA replication, DNA strand breaks caused by abortive topoisomerase I and topoisomerase II activity, or from ROS produced from normal metabolic byproducts that can attack DNA. Exogenous sources mainly include mutagenic chemicals, ultraviolet and ionizing radiations (IR). Cell cycle checkpoints are capable of detecting the DNA lesions, signal their presence, and activate pathways that delay cell cycle progression, repair the DNA lesions, or eliminate the genetically unstable cells by inducing cell death [18–23].

At the core of the DNA damage response (DDR) signaling in mammalian cells are the protein kinases Ataxia telangiectasia mutated (ATM) and ATM- and RAD3-related (ATR). ATM and ATR phosphorylate and activate two other kinases, CHK1 and CHK2 which, together with ATM and ATR, are the master regulator of the cell-cycle checkpoints [24]. By regulating the activity of CDKs, these molecules slow down or arrest the cell cycle progression at the G_1_–S, intra–S and G_2_–M phases to allow DNA damage to be repaired. Concomitantly, ATM and ATR promote DNA repair by controlling the expression, activity, or recruitment of diverse factors to sites of DNA damage. Generally, the DDR machinery is able to repair the DNA damage that a cell may accumulate during its life cycle, but if the extent of the damage is deemed too high, cell death by apoptosis or cellular senescence is induced [25, 26].

In our previous work, we discovered that ATM phosphorylates PTEN at position 398 (threonine in human; serine in mouse) upon activation of the DDR [27]. To understand the biological implications of PTEN phosphorylation by ATM, we generated a mouse model that constitutively expresses a mutated form of PTEN that cannot be phosphorylated by ATM, substituting serine for alanine at position 398 (PTEN-398A). Expression of PTEN-398A caused acceleration of tumor development and progression in a well-established mouse model of HER2-positive brea`st cancer [28]. Using a molecular biology approach, we identified a novel mechanism by which the phosphorylation of PTEN by ATM regulates its cellular redistribution and contributes to the tumor suppressor function of the protein.

## Materials and methods

### Construction of *Pten*^*398A*^ targeting vector

A targeting construct was designed to replace the last exon of *Pten* with a version in which serine 398 was mutated to alanine along with the creation of a silent *SphI* restriction site. To generate the short-arm and long arm genomic targeting fragments, we designed primers based on mouse *Pten* genomic sequence (GenBank Accession No. NC_000085) to use in polymerase chain reaction (PCR) from mouse 129J genomic DNA (Jackson Laboratory, Bar Harbor, Maine). In brief, PCR primers 5′-AGT CGA CAT GTG CCT CAA TGC TTG TCT AAC ATG AGA AAT GC-3′ (*Pten SalI* sense) and 5′-CCT CGA GAA GCT TAT AAT TCT ATA AAA GTG CAA ACT GAA GGC AAT G-3′ (*Pten HindIII* antisense) were used to amplify a 978 bp short-arm fragment (corresponding to *mPten* intron 8) from 200 ng of 129J genomic DNA using the Expand^™^ High Fidelity PCR system (Roche). The Expand^™^ Long Template PCR system (Roche GmbH, Germany) was used to amplify a 4.2 kb long arm fragment (corresponding to the entire *mPten* exon 9 coding region and part of the 3′UTR) from 129J Genomic DNA using the PCR primers 5′-TGC GGC CGC GAA TTT TGT GAT TTA GTG AGT TCA TTG CCT TCA G-3′ (*Pten NotI* sense) and 5′-GCG GCC GCC TAC TAC TCT GGA CAA GTC CCG ATG AAA CC-3′ (Pten *NotI* antisense). The resulting PCR products were gel-purified using the Nucleospin^®^ Gel and PCR Clean-up system (Machery-Nagel Gmbh & Co., Germany), TA-cloned into pCR2.1-TOPO (Invitrogen, San Diego, CA), and subcloned into a modified pBluescript II KS (Stratagene, La Jolla, CA) vector containing a PGK-neomycin cassette flanked by both FRT and LOXP sequences. A diphtheria toxin (*DTA*) gene was inserted 3′ of the long arm to negatively select against non-homologous targeting. Insert sequence was validated using fluorescent dideoxy-nucleotide sequencing and automated detection (ABI/Perkin Elmer, Forest City, CA).

### *Pten*^*398A*^ mutagenesis

To mutate serine at position 398 of *Pten*, a S to A mutation of amino acid 398 and silent *SphI* restriction site was introduced in exon 9 of the 3′ long arm by changing 1 basepair (TCA to GCA) by site-directed mutagenesis with the Quick Change mutagenesis II ^TM^ system (Stratagene, La Jolla, CA). The primers used for creation of the 398A and silent *SphI* site were: *PTEN_398A_U1*: 5′-GAA CCT TTT GAT GAA GAT CA*G CAT* *GC*A CAA ATT ACA AAA GTC TG-3′ and *PTEN_398A_L1:* 5′-CAG ACT TTT GTA ATT TGT GCA TGC TGA TCT TCA TCA AAA GGT TC-3′. The PCR-generated mutagenized fragment was completely sequenced in order to verify the sequence.

### Targeted disruption of the murine *Pten* gene in ES cells

The *Pten*^*398A*^ targeting vector (25 μg) was linearized with *SalI* restriction endonuclease at the short arm and electroporated into E14 ES cells (derived from the R129J strain) using a Bio-Rad Gene Pulser, 0.34 kV, and 0.25 mF. ES cell culture was carried out as previously described [29]. After G418 selection (250 µg/ml), homologous recombinants were identified by 5′ and 3′ flanking PCR and confirmed by Southern blot analysis and sequencing following published protocols [29]. Homologous recombination (HR) at the 5′ short-arm was confirmed by Terra™ PCR Direct (Takara Bio, Mountain View, CA, USA) amplification of a 1680 bp fragment using the primers mPten Ex8 Sense: 5′-GCA AAC AAA GAC AAG GCC AAC CGA TAC TTC-3′, in exon 8 of *Pten*, and the vector-specific primer *KI_5_PCR_antisense*: 5′-CGT CAA CCA AGC TCT GAT AGA GTT GCT CAA GG-3′. HR at the 3′ long-arm and sequence validation of the 398A was confirmed by Terra^™^ PCR Direct amplification of a 4395 bp fragment using the vector-specific primer *KI_3_PCR_sense*: 5′-GAG TGC GAT CTA GCC AGA CGA GGG TTC-3′ and the *Pten* 3′UTR primer: *PTEN_UTR_antisense* 5′-CAA CTT CAT GTA ACA TTA AGA CTC CAC ATT GAC-3′.

HR of the targeting vector with the endogenous locus results in insertion of a novel *HindIII* site into the *Pten* locus, thus allowing the targeted and wild-type alleles to be distinguished by Southern analysis with the 867 bp 5′ flanking genomic probe (5′ Probe). The ~9 kb *HindIII* fragment corresponding to the wild-type allele is decreased to ~4.5 kb upon disruption of the *Pten* locus by integration of the Neomycin resistance gene. The 5′ Flanking Probe was generated by PCR using the primers 5′-GCT GCT AGA GTC TAG TCT TAG AAC TTA CTG TTT G-3′ (5′Probe sense) and 5′-TAC TAT TAC TTC TTC ACA ACC ACT TCT TTC AAC-3′ (5′Probe antisense). Genomic DNA was digested with *HindIII*, resolved on an agarose gel, transferred to Hybond N+ membrane, and hybridized to ^32^P labeled *Pten* 5′ Probe.

### Generation of *Pten*^*S398A*^ mice

Chimeric mice were produced by microinjection of independent *Pten*^*S398A*^ ES cell clones into E3.5 C57BL/6J blastocysts and transferred to ICR pseudopregnant foster mothers. Chimeric males were mated with C57BL/6J females (Jackson Laboratory). Germ line transmission of the mutant allele was confirmed by PCR and Southern blot analysis of tail DNA from mice with an agouti coat color. The PGK-Neo cassette was removed by crossing with Flp-deleter mice (Jackson Laboratory stock #009086) [30] and PCR genotyping and sequence validation of recombination at the FRT sites.

All experimental procedures strictly adhered to the Canadian Council on Animal Care guidelines.

### Antibodies

The following antibodies were purchased from Cell Signaling Technology: PTEN (138G6) (#9559), Akt (#9272), phospho-Akt (Ser473) (193H12) (#4058). Phospho-p27^Kip1^ (phospho T157) was purchased from abcam (ab85047). Phospho-Histone H2A.X (Ser139) (JBW301) (05-636) was from Millipore. Antibody against 53BP1 (A300-272A) was from Bethyl Laboratories.

### Cell culture, viral infections, and reagents

MCF10A PTEN-398A and isogenic MCF10A PTEN-WT cells were maintained in DMEM/F-12 (1:1), supplemented with 10% fetal bovine serum (Gibco), Pen/Strep (100 mg/ml), insulin (5 μg/ml), hydrocortisone (1 μg/ml) (all from Sigma), and EGF (5 ng/ml, Peprotech).

For expression in MCF10A cells, *PTEN* and *PTEN-398A* were cloned into pBabe-Flag as described in Bassi et al. [27]. Viral transduction with retroviruses expressing FLAG-PTEN or FLAG-PTEN-398A was performed in MCF10A cells by following the procedures described previously [31]. Z-VAD-FMK was from MedKoo Biosciences (Catalog #533011).

### Cell lysis, immunoblotting, and immunoprecipitations

Unless indicated otherwise, for immunoblotting cells were lysed in Laemmli sample buffer (60 mM Tris-Cl pH 6.8, 2% sodium dodecyl sulfate, 10% glycerol, 5% β-mercaptoethanol), normalized for total protein content, resolved by sodium dodecyl sulphate-polyacrylamide gel electrophoresis, and transferred to PVDF membranes (Millipore). Membranes were blocked in Odyssey^®^ Blocking Buffer (LI-cor) and probed with the indicated antibodies.

For immunoprecipitations, cells were lysed for 30 min on ice in IP buffer (150 mM NaCl–50 mM Tris pH 7.4–10% glycerol–1% Triton-X 100), supplemented protease inhibitor cocktail (Sigma). Insoluble material was removed by centrifugation at 15,000 × *g* for 15 min at 4 °C. Immunoprecipitations were performed by gentle rotation overnight at 4 °C, and then immune complexes were washed four times in cold IP buffer and resuspended in Laemmli buffer.

### Immunofluorescence

Cells cultured on glass cover slips were rinsed in phosphate-buffered saline (PBS), fixed with 3.7% formaldehyde in PBS for 10 min at room temperature, permeabilized with PBS plus 0.5% Triton X-100 for 5 min, blocked overnight at 4 °C with PBS containing 1% bovine serum albumin (Fisher), and then incubated with primary antibodies (PTEN Cell signaling 9559, 1:200; 53BP1 Bethyl A300-081A 1:500; γH2AX Millipore 05-636 1:1000). After 3 washes with PBS, 5 min each, at room temperature, samples were incubated for 30 min with a 1:400 dilution (in PBS) of goat anti-rabbit or anti-mouse IgG conjugated to the fluorescent Alexa 488 dye,goat anti-mouse IgG conjugated to the fluorescent Alexa 546 dye, or donkey anti-rabbit conjugated to the fluorescent Alexa 647 dye (Invitrogen Molecular Probes), washed three times, stained with DAPI and mounted in Mowiol.

### Cell proliferation assays

Cell number was assessed indirectly by using the Sulforhodamine B (SRB) assay [32].

### HR assays

HR and NHEJ assay were performed as described in Weinstock et al. [33] MCF10A cells (PTEN 398A and PTEN WT) were stably transduced with differentially mutated green fluorescent protein (GFP) and with pCMV3xnlsI-SceI, using Amaxa^™^ Nucleofector^™^ Technology (Lonza). At 2 days post transfection, GFP signals were acquired using a FACSCanto II flow cytometer instrument and analyzed using FACSDiva and FlowJo software (BD Biosciences). Recombination efficiency was calculated as the number of GFP-positive cells in the samples divided by the number of GFP-positive cells in MCF10A PTEN-WT cells. For each experiment, 10,000 cells were scored per treatment group.

### Cell cycle analysis

Cell cycle analysis was carried out by flow cytometry. Briefly, MCF10A cells were seeded into 6-well culture plates, treated as indicated, collected, fixed, stained with propidium iodide (100 µg/mL) and RNAse (20 µg/mL) in PBS for 1 h, acquired on a FACSCanto II flow cytometer instrument and analyzed using FACSDiva and FlowJo software (BD Biosciences). Similarly, indirect immunofluorescence on ethanol-fixed MCF10A cells (70% in PBS) was performed to quantify mitotic cells using an anti-phospho-H3 (Ser10) specific antibody (CellSignaling) detected by an Alexa488-labeled goat anti-rabbit secondary antibody (Invitrogen). Cells were acquired on a FACSCanto II flow cytometer instrument and analyzed using FACSDiva and FlowJo software (BD Biosciences). Ten thousand events were analyzed for each sample.

### Annexin V/7-AAD assay for apoptosis

For Annexin V/7-AAD assays, cells were stained with Annexin V–FITC and 7-AAD, and evaluated for apoptosis by flow cytometry according to the manufacturer’s protocol (BD PharMingen, San Diego, CA, USA). Briefly, 1 × 10^5^ cells were washed twice with PBS, and stained with 5 μl of Annexin V–FITC and 10 μl of &-AAD (5 μg/ml) in 1× binding buffer (10 mM HEPES, pH 7.4, 140 mM NaOH, 2.5 mM CaCl_2_) for 15 min at room temperature in the dark. The stained cells were acquired on a FACSCanto II flow cytometer instrument and analyzed using FACSDiva and FlowJo software (BD Biosciences). Ten thousand events were analyzed for each sample.

## Results

### Pten^398A^ accelerates MMTVneu-driven mammary tumorigenesis

**Fig. 1:**
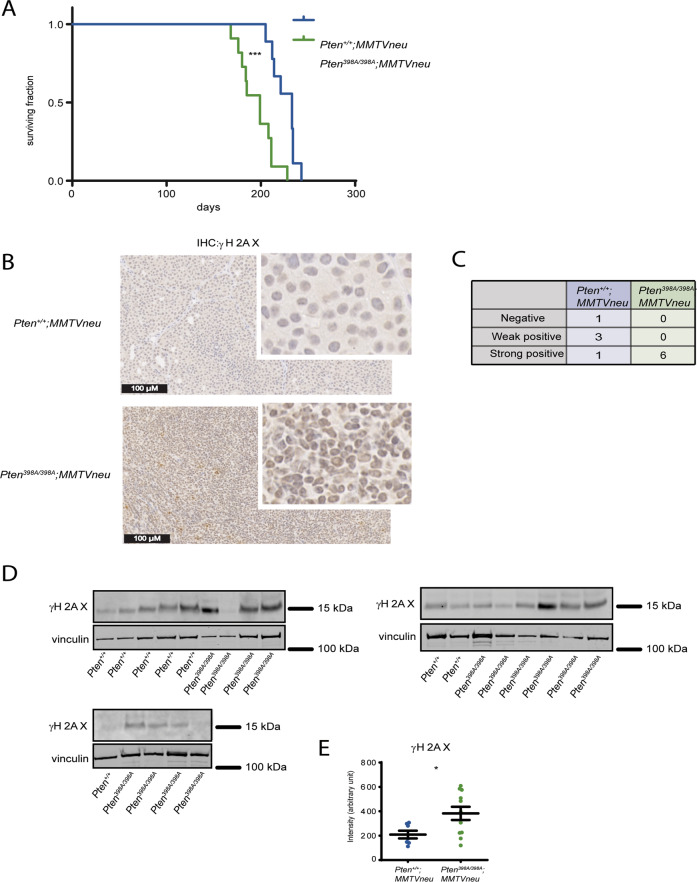
Characterization of *Pten*^*398A/398A*^*;MMTVneu* knock-in mouse. **A** Survival rate in *Pten*^*+/+*^*;MMTVneu* and *Pten*^*398A/398A*^*;MMTVneu* mice (*n* = at least 10 mice/group). (***p = 0.0005; Log-rank test for comparisons of Kaplan–Meier survival curves). **B** Representative sections of *Pten*^*+/+*^*;MMTVneu* and *Pten*^*398A/398A*^*;MMTVneu* mammary tumors stained with a γH2AX antibody (magnification, ×10; main image zoom, ×20, insets zoom ×80). **C** Semi-quantitatively expression scoring of Gamma-H2AX IHC stained tumors from *Pten*^*+/+*^*;MMTVneu* and *Pten*^*398A/398A*^*;MMTVneu* mice. **D** Immunoblot analysis of γH2AX in *Pten*^*+/+*^*;MMTVneu* and *Pten*^*398A/398A*^*;MMTVneu* mammary tumor samples. Vinculin was used as a loading control. **E** Quantification of γH2AX levels, normalized to vinculin, from the blots shown in panel (**E**) (*t* test, *p = 0.0332).

To study the in vivo function of PTEN phosphorylation by ATM, we engineered a knock-in allele in mice, in which serine at position 398 is substituted for alanine (*Pten*^*398A*^) (Supplementary Fig. 1A, details in materials and methods section). *Pten*^*398A/+*^ and *Pten*^*398A/398A*^ mice were viable, and their development was overtly normal. To investigate a possible role for ATM-dependent PTEN phosphorylation in breast cancer, we bred the *Pten*^*398A*^ allele on the background of mice expressing the inactivated *neu* (*Erbb2*) fusion gene under the transcriptional control of the mouse mammary tumor virus (MMTV) promoter/enhancer [34] (*MMTVneu*, Supplementary Fig. 1B). In this well-established model, the *MMTVneu* transgene is expressed at low levels in normal mammary epithelium, leading to the development of tumors in the mammary glands with a reported median incidence of 205 days [34]. In our hands, *Pten*^*+/+*^*;MMTVneu* mice developed mammary tumors with the expected latency and displayed a median survival of 233 days (Fig. 1A). Comparatively, tumor development was accelerated in *Pten*^*398A/398A*^*;MMTVneu* mice, in which median survival was reduced to 199 days (*p* < 0.001 by Mantel–Cox or Gehan–Breslow–Wilcoxon test) (Fig. 1A). Histological and immunohistochemical (IHC) analyses indicated that the tumors expressed strong levels of HER2, and exhibited the expected morphology and differentiation pattern [35] (Supplementary Fig. 2A, B).

Because ATM is an important protein for the control of the DDR, we hypothesized that accelerated tumorigenesis in *Pten*^*398A/*^^*398A*^*;MMTVneu* mice could be associated with increased genomic instability. Phosphorylation of histone H2A variant H2AX at Ser 139 (γH2AX) can be used as a marker of DNA damage in cells [36]. Therefore, we examined γH2AX by IHC on paraffin-embedded tumor samples obtained from *Pten*^*+/+*^*;MMTVneu* and *Pten*^*398A/398A*^*;MMTVneu* mice. γH2AX immunoreactivity was scored using a semi-quantitative method (Table 1). *Pten*^*+/+*^*;MMTVneu* tumors generally showed little γH2AX expression (Fig. 1B). By contrast, all the *Pten*^*398A/398A*^*;MMTVneu* samples analyzed were strongly positive for γH2AX, suggesting higher levels of genomic instability (Fig. 1B, C). We confirmed these results by western blot analyses of tumor samples, which indicated that *Pten*^*398A/398A*^*;MMTVneu* tumors expressed higher levels of γH2AX than their *Pten*^*+/+*^*;MMTVneu* counterparts (Fig. 1D, E). We assessed the proliferation index of the tumors, by performing IHC for Ki-67, a marker widely used in routine pathology. No significant difference in Ki67 immunoreactivity was detected between tumors of the two genotypes *Pten*^*+/+*^*;MMTVneu* and *Pten*^*398A/398A*^*;MMTVneu* (Supplementary Fig. 2C, D).

**Table. 1:** Criteria for the semi-quantitative evaluation of γH2AX staining by immunohistochemistry on mouse mammary tumors.

Negative	Weak punctate staining encompassing <50% of the nucleus of <50% of neoplastic cells
Weak positive	Moderate to strong punctate staining encompassing <50% of the nucleus of 50–75% of the neoplastic cells
Strong positive	Moderate to strong punctate staining encompassing up to 90% of the nucleus of >75% of neoplastic cells

These results indicate that the *Pten*^*398A*^ mutation accelerates *MMTVNeu*-driven tumorigenesis, and that this is associated with higher genomic instability but no evident alterations in cell proliferation.

### Blocking ATM-dependent PTEN phosphorylation causes genomic

To mechanistically dissect how phosphorylation of PTEN by ATM regulates the DDR, we turned to more tractable cellular models. We generated MEFs from *Pten*^*398A/398A*^ and *Pten*^*+/+*^ littermates. Western blot analysis indicated that PTEN protein levels were not affected by the 398A mutation (Supplementary Fig. 3A, B). Furthermore, levels of phosphorylated AKT, a marker of PI3K pathway activity, were similar in *Pten*^*398A/398A*^ and *Pten*^*+/+*^ MEFs (Supplementary Fig. 3B). We also generated a non-transformed human mammary epithelial cell line (MCF10A) [37] stably expressing wild-type PTEN (PTEN-WT), or the mutant form of PTEN that cannot be phosphorylated by ATM (PTEN-398A). In these cells, the endogenous *PTEN* gene has been deleted using CRISPR/Cas9 editing, creating PTEN null cells which were then reconstituted using stable transduction with either the wild type or the mutant form of the protein. The levels of PTEN protein and phosphorylated AKT were similar in MCF10A cells expressing PTEN-WT and PTEN-398A (Supplementary Fig. 3A), making these cells a suitable alternative model for the study of the molecular role of the phosphorylation of PTEN by ATM.

**Fig. 2:**
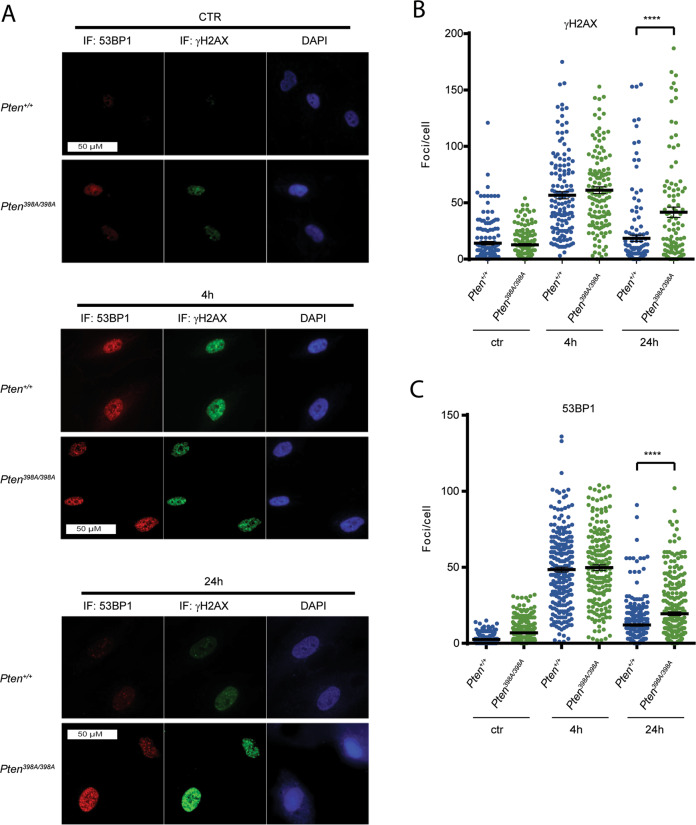
Defective repair of DNA damage in cells expressing PTEN 398A. **A** Representative images of *Pten*^*+/+*^ and *Pten*^*398A/398A*^ MEF cells exposed or not (CTR) to 10 Gy of ionizing radiation, fixed at the indicated times post-IR, and immunostained for γH2AX, and 53BP1. DAPI was used to stain DNA. **B** Quantification of the number of γH2AX nuclear foci in MEF cells expressing PTEN WT or PTEN 398A. Nuclear foci in at least 100 nuclei were counted. (*t* test, ****denotes *p* < 0.0001. **C** Quantification of the number of 53BP1 nuclear foci in *Pten*^*+/+*^ and *Pten*^*398A/398A*^ MEF cells. Mean and standard error of the mean are indicated for each transfection. Nuclear foci in at least 100 nuclei were counted. ****denotes *p* < 0.0001.

*Pten*^*+/+*^ and *Pten*^*398A/398A*^ MEFs were subjected to a dose of 10 Gy IR, and their proficiency in repairing the DNA damage was assessed by measuring the number of γH2AX and 53BP1foci per cell. In *Pten*^*+/+*^ MEFs, we observed a peak in the number of foci per cells at 4 h after irradiation, returning near baseline levels by 24 h (Fig. 2A–C). *Pten*^*+/+*^ and *Pten*^*398A/398A*^ accumulated similar numbers of foci per cell at 4 h post-IR. However, *Pten*^*398A/398A*^ MEFs retained higher numbers of foci than their *Pten*^*+/+*^ counterparts at the 24 h time point, suggesting reduced DNA repair capability (Fig. 2A–C). Similar results were obtained when comparing MCF10A cells stably expressing PTEN-WT vs. PTEN-398A (Supplementary Fig. 4A–C). To assess whether the impaired ability to resolve DNA damage foci in cells expressing PTEN-398A is due to defects in specific DNA repair pathways, we evaluated the efficiency of HR and non-homologous end-joining (NHEJ), using reporter assays. As previously reported [38], PTEN-WT expression enhanced the efficiency of both HR and NHEJ (Supplementary Fig. 4D, E). In MCF10A cells, PTEN-398A had similar effects to PTEN-WT in these experiments. These results indicate that the accumulation of DNA damage in cells expressing the mutated form of PTEN is not due to intrinsic defects in the DNA repair machinery (Supplementary Fig. 4D, E).

### PTEN-S398A mutation induces resistance to apoptosis

To further understand how blocking ATM-dependent PTEN phosphorylation affects the cellular response to genotoxic insult, we evaluated the activity of stress response and apoptotic pathways in lysates from MCF10A cells expressing PTEN-WT or PTEN-398A, using an antibody array. As expected, cells expressing PTEN-WT promptly induced multiple stress response and apoptosis markers, as early as 30 min following genotoxic treatment (IR) (Supplementary Fig. 5A). In contrast, cells expressing PTEN-398A cells showed a more modest induction of the same markers (Supplementary Fig. 5B), consistent with defective activation of the stress and apoptotic programs.

**Fig. 3:**
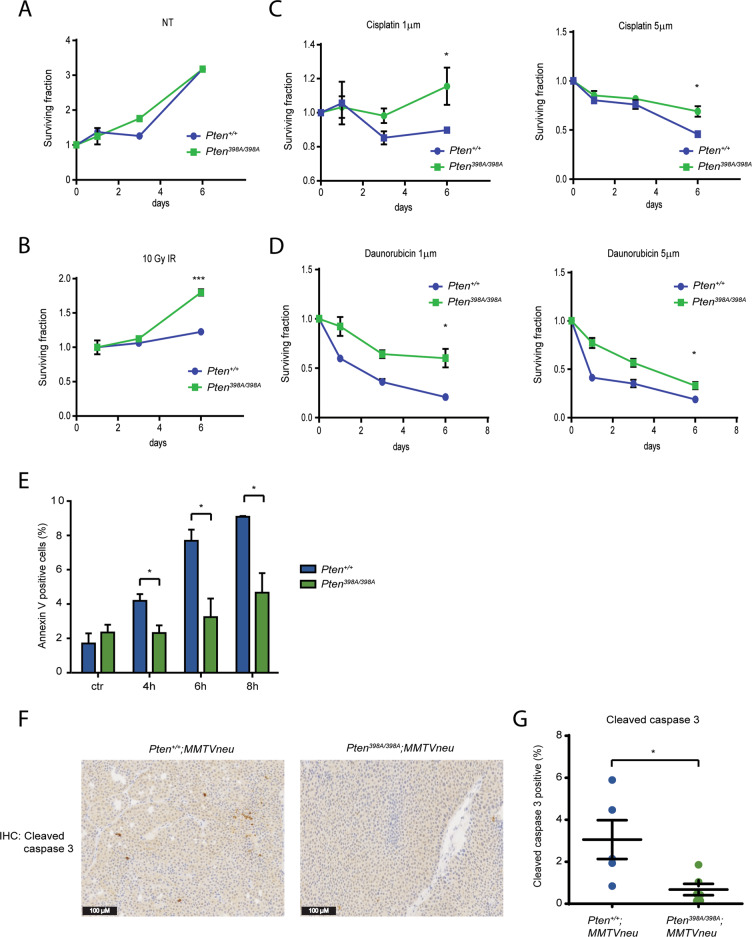
Resistance to genotoxic stress and defective activation of apoptosis induced by expression of PTEN 398A. **A**–**D** Surviving fraction of *Pten*^*+/+*^ and *Pten*^*398A/398A*^ MEFs, recorded over 6 days, in untreated cells (**A**) or following exposure to 5 Gy of ionizing radiation (**B**), 1 and 5 µm Cisplatin (**C**) and 1 and 5 µM Daunorubicin (**D**). All data are expressed as mean ± SD. Experiments in triplicate. *t* test, *p < 0.05. **E** Quantification of annexin-V/FITC positive *Pten*^*+/+*^ and *Pten*^*398A/398A*^ MEF cells after treatment with 10 Gy of ionizing radiation. Cells were collected at the indicated time points after treatment, stained and analyzed by flow cytometry. Data are expressed as mean ± SD. *p < 0.05 compared with corresponding controls (*n* = 3). **F** Immunohistochemical staining of cleaved cleaved caspase 3 in *Pten*^*+/+*^*;MMTVneu* and *Pten*^*398A/398A*^*;MMTVneu* mammary tumor samples. **G** Quantification of cleaved cleaved caspase 3 positive cells in tumor sections from *Pten*^*+/+*^*;MMTVneu* and *Pten*^*398A/398A*^*;MMTVneu* mammary tumors (*t* test, *p = 0.0402).

We reasoned that increased genomic instability, coupled with the defective activation of the stress response and apoptotic pathways, in cells expressing the mutant form of PTEN should lead to phenotypic differences in response to DNA damaging agents. We therefore assessed the resistance of cells expressing wild-type or mutant PTEN to genotoxic stress. There was no difference in the growing rate of *Pten*^*+/+*^ and *Pten*^*398A/398A*^ MEF cultures, or of MCF10A cells expressing PTEN-WT vs. PTEN-398A, under normal culture conditions (Fig. 3A and Supplementary Fig. 5C). The cells were then subjected to different genotoxic stress treatments: IR, cisplatin, and daunorubicin. In all the conditions, *Pten*^*398A/398A*^ MEFs and PTEN-398A-expressing MCF10A cells were more resistant to genotoxic stress than their respective counterparts expressing wild-type PTEN (Fig. 3B–D and Supplementary Fig. 5D, E). Consistent with this phenotype, apoptosis induction by IR was lower in MCF10A cells expressing PTEN-398A vs. PTEN-WT, as judged by Annexin V staining using flow cytometry (Fig. 3E).

We confirmed these observations in our in vivo model by performing immunohistochemistry staining with caspase-3 antibody, a widely used marker for detecting apoptotic cells, on *Pten*^*+/+*^*;MMTVneu* and *Pten*^*398A/398A*^*;MMTVneu* tumors. Indeed, *MMTVneu* tumors harbored fewer caspase-3-positive cells when compared to their corresponding *Pten*^*+/+*^*;MMTVneu*, *Pten*^*398A/398A*^ (Fig. 3F, G). Altogether, these results suggest that cells in which PTEN cannot be phosphorylated by ATM are resistant to the induction of apoptosis following genotoxic stress. To assess whether the persistence of cells expressing PTEN-398A with unresolved DNA damage following irradiation (Fig. 2B, C and Supplementary Fig. 4B, C) is a consequence of the failure to induce the apoptotic response, we pretreated cells with z-VAD-FMK (zVAD), a pan-caspase inhibitor, prior to IR. zVAD increased the number of unresolved DNA damage foci in PTEN-WT cells—compared to that of DMSO treatment—to a level comparable to that observed in vehicle (DMSO)-treated PTEN-398A cells (Supplementary Fig. 5F). However, it has to be noted that zVAD pretreatment also slightly increased the number of unresolved DNA damage foci in PTEN-398A cells following IR.

### Impaired G1/S cell cycle checkpoint in cells deficient for ATM-dependent PTEN phosphorylation

To complement the above studies, we performed cDNA microarray analyses on MCF10A cells expressing either PTEN-WT or PTEN-398A, and treated with cisplatin to induce genotoxic stress. The list of differentially expressed genes was analyzed by Gene Set Enrichment Analysis [39]. Interestingly, genes upregulated in cells expressing PTEN-398A were related to the activation of the G1/S checkpoint, such as G1/S phase-specific transcription, E2F targets and cell cycle regulatory genes controlled by the Rb1 pathway (Supplementary Fig. 6A).

**Fig. 4:**
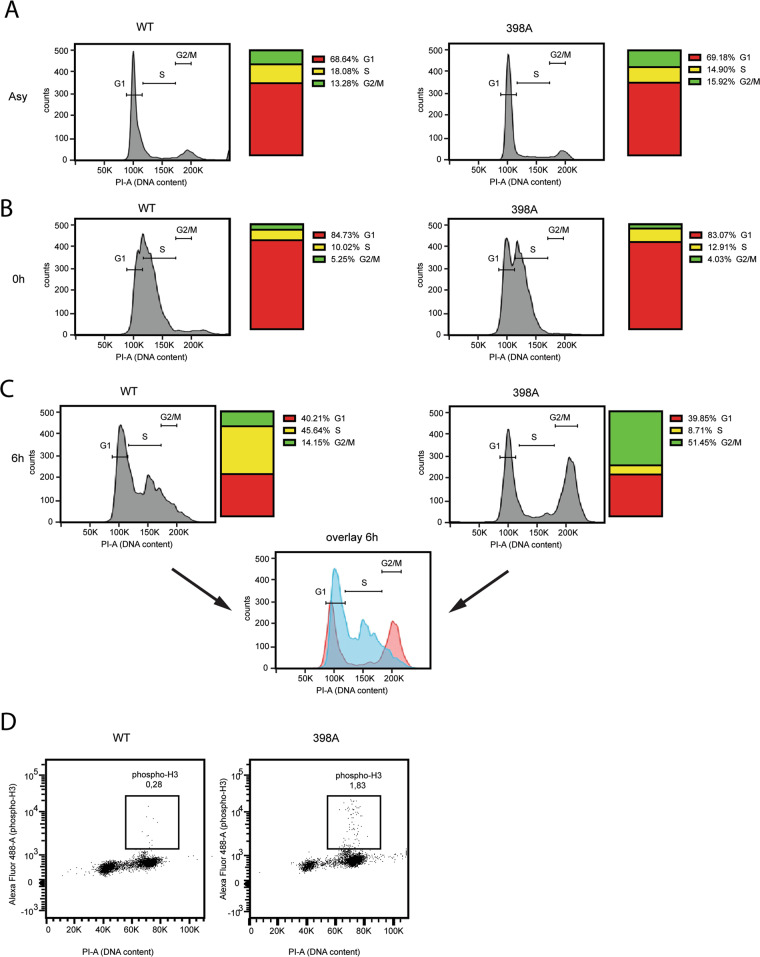
Altered cell cycle progression following DNA damage exposure in cells expressing PTEN 398A. **A**–**C** Cell cycle profiles, assessed by flow cytometry, of MCF10A cells expressing PTEN-WT or PTEN-398A. Cells were either untreated or treated with IR (5 Gy). The captions (and corresponding quantification on the right) correspond to asynchronous cells (asy, **A**), cells arrested by double thymidine block (0 h, **B**) and cells synchronized by double thymidine block, released in full media, treated with IR and collected after 6 h (**C**). The experiment was repeated 3 times; a representative example is shown. **D** Phospho-H3 and DNA content, assessed by flow cytometry, in MCF10A cells expressing PTEN-WT or PTEN-398A that were synchronized by double thymidine block, released in full media, treated with IR and collected after 6 h. The figure is representative of three different experiments.

Based on the above observations, we speculated that the resistance of cells expressing PTEN-398A to genotoxic stress may be due to failure to arrest the cell cycle in response to DNA damage. To test this possibility, we measured the ability of synchronized cells expressing wild-type or mutant PTEN to undergo cell cycle arrest at the G1/S checkpoint, in response to DNA damage. No noticeable difference in the proportion of cells in G1, S, and G2/M phases was observed in normally cycling cells expressing PTEN-WT or PTEN-398A (Fig. 4A). To synchronize the cells at the G1/S checkpoint, we subjected them to a double thymidine pulse. This treatment effectively arrested both PTEN-WT and PTEN-398A cells at the G1/S boundary (Fig. 4B). The cells were then released in normal growth media, treated with IR during S phase progression, and collected 6 h later for cell cycle distribution analysis by flow cytometry (following an experimental scheme illustrated in Supplementary Fig. 7A). While most of the PTEN-WT cells were arrested in S-phase, PTEN-398A cells failed to arrest the cell cycle, having instead progressed through the G2/M phase (Fig. 4C). This observation was confirmed by measuring the proportion of cells expressing Phospho-Histone H3 (Ser10), which marks cells in the mitotic phase. (Fig. 4D). There was no difference in cell cycle progression in non-irradiated cells expressing PTEN-WT vs. PTEN-398A following release from the double thymidine block (Supplementary Fig. 7B).

**Fig. 5:**
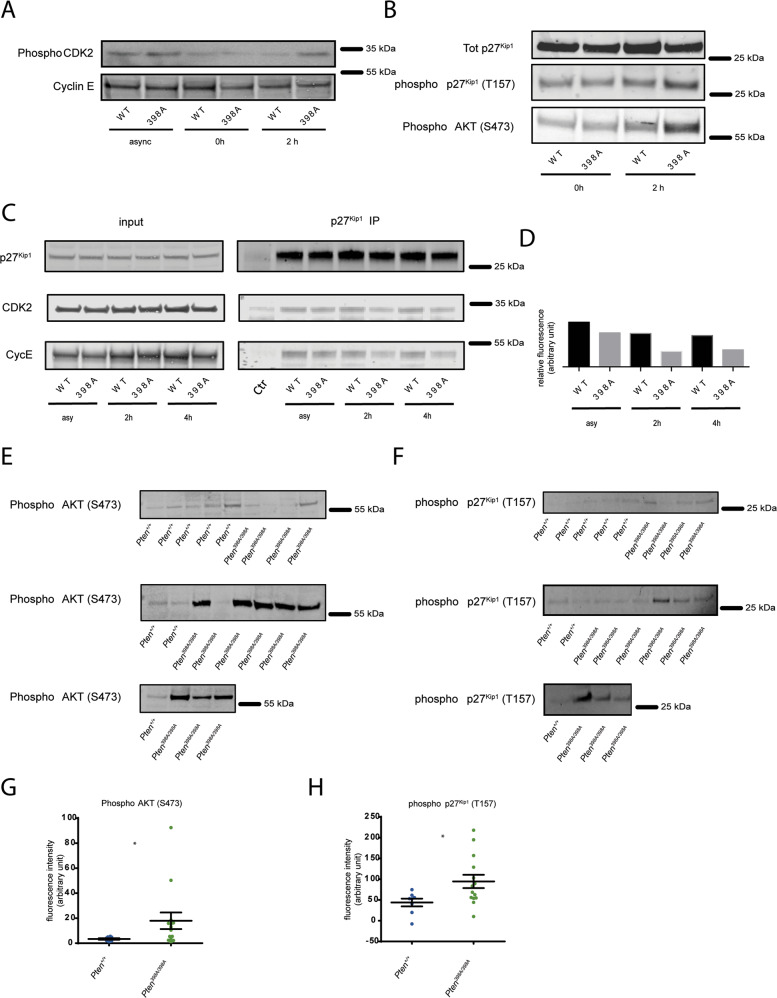
Cells expressing PTEN 398A display defective activation of the G1/S checkpoint. **A** Levels of phosho-CDK2 and CycE in lysate prepared from MCF10A cells expressing PTEN-WT or PTEN-398A, either grown asynchronously (asy), arrested by double thymidine block (0 h) or synchronized by double thymidine block, released in full media, treated with IR and collected after 6 h. **B** Levels of p27^Kip1^, phospho-T157 p27^Kip1^, or phosho-S473 AKT in lysates prepared from MCF10A cells expressing PTEN-WT or PTEN-398A. Cells were either arrested by double thymidine block (0 h) or synchronized by double thymidine block, released in full media, treated with IR and collected after 2 h. **C** Levels of p27^Kip1^, CDK2, or CycE in total cell lysates (input), or in p27^Kip1^ immunoprecipitates (p27 IP) from MCF10A cells expressing PTEN-WT or PTEN-398A. Cells were either grown asynchronously (asy) or synchronized by double thymidine block, released in full media, treated with IR and collected after 2 or 4 h. **D** Quantification of CDK2 binding to p27^Kip1^ from the immunoprecipitation experiment shown in panel (**G**). Values (in arbitrary unit) are expressed as signal of co-immunoprecipitated CDK2 relative to the signal of immunoprecipitated p27^Kip1^. **E** Immunoblot analysis of phospho-AKT in *Pten*^*+/+*^*;MMTVneu* and *Pten*^*398A/398A*^*;MMTVneu* mammary tumor samples. Vinculin (shown in Fig. 1D) was used as a loading control. **F** Immunoblot analysis of phospho-p27^Kip1^ in *Pten*^*+/+*^*;MMTVneu* and *Pten*^*398A/398A*^*;MMTVneu* mammary tumor samples. Vinculin (shown in Fig. 1D) was used as a loading control. **G** Quantification of phospho-AKT (S473) levels, shown in panel (**E**), normalized to vinculin, shown in Fig. 1D. **D** Quantification of phospho-p27^Kip1^ (T157) levels, shown in panel (**F**), normalized to vinculin, shown in Fig. 1D.

At the G1/S-phase checkpoint, CDK2 becomes hypo-phosphorylated [40]. Accordingly, cells expressing PTEN-WT, that were arrested in S phase following release from double thymidine block and irradiation, exhibited low levels of CDK2 phosphorylation (Fig. 5A). Conversely, PTEN-398A cells subjected to the same treatment had higher levels of phosphorylated CDK2 (Fig. 5A), consistent with defective activation of the DNA damage checkpoint.

p27^Kip1^, a Cip/Kip member, is a CDK inhibitor that causes G1/S arrest by inhibiting the activity of the CDK2-Cyclin E complex [41]. Phosphorylation of p27^Kip1^ by AKT reduces its inhibitory activity towards CDK2–Cyclin E complex and promotes cell cycle progression [42, 43]. Following the above protocol to synchronize and release cells in G1/S (Supplementary Fig. 7A), we evaluated the activation of the AKT/p27^Kip1^ axis 2 h after IR. At that time point, levels of phosphorylated AKT and phosphorylated p27^Kip1^ were higher in cells expressing PTEN-398 compared to their PTEN-WT counterparts (Fig. 5B). Given that p27^Kip1^ binds CDK2/Cyclin E to inhibit cell cycle progression, we sought to determine the composition of this complex under our experimental conditions. We immunoprecipitated endogenous p27^Kip1^ from cells expressing PTEN-WT and PTEN-398A, and immunoblotted for CDK2 and Cyclin E. While p27^Kip1^ effectively co-immunoprecipitated with CDK2 and Cyclin E in irradiated PTEN-WT cells, these interactions were diminished in PTEN-398A cells (Fig. 5C, D).

To verify the relevance of our findings in the context of *MMTVNeu* model, we evaluated the activation of the AKT-p27^Kip1^ axis in *Pten*^*+/+*^*;MMTVNeu* and *PTEN*^*398A/398A*^*;MMTVNeu* mammary tumors. Consistently with what we observed in MCF10A cells, immunoblotting analyses revealed higher levels of phosphorylated AKT and phosphorylated p27^Kip1^ in lysates from *PTEN*^*398A/398A*^*;MMTVNeu* tumors compared with their *Pten*^*+/+*^*;MMTVNeu* counterparts (Fig. 5E–H). This observation is of particular importance as it establishes a direct causal link between the molecular effects elicited by PTEN-398 mutation and the in vivo mammary tumor phenotype.

Altogether, our results indicate that preventing phosphorylation of PTEN by ATM increases AKT and p27^Kip1^ activation following DNA damage, in turn favoring the activity of the CDK2/Cyclin E complex, and driving cell cycle progression.

### Blocking ATM-dependent phosphorylation impairs subcellular redistribution of PTEN following DNA damage

**Fig. 6:**
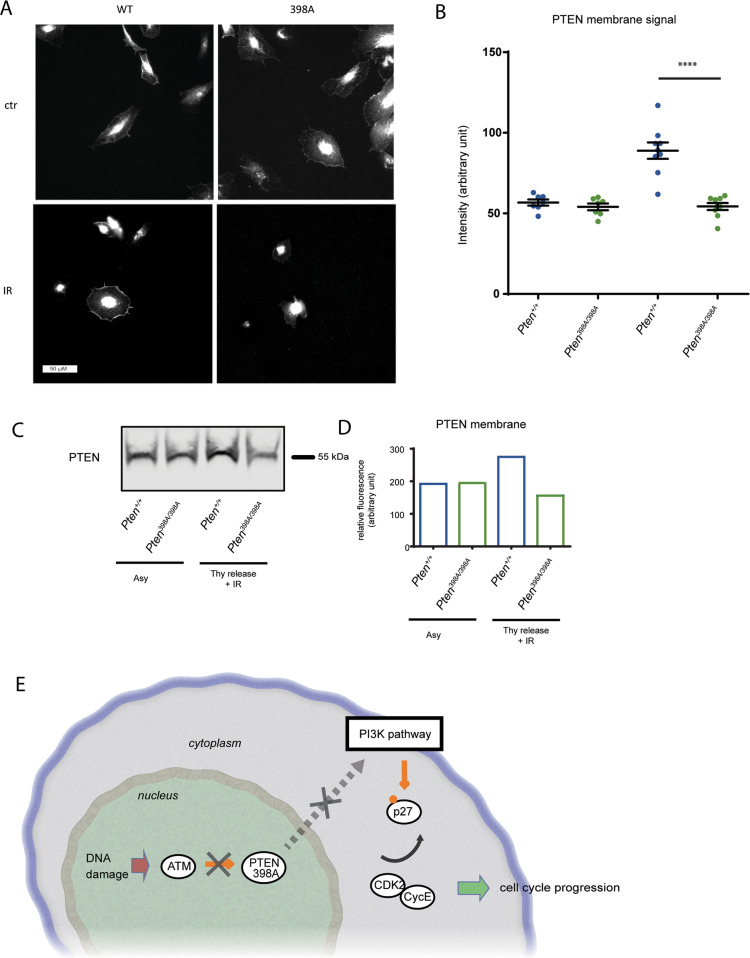
Phosphorylation by ATM of PTEN in position 398 mediates its relocalization to the membrane. **A** Representative images of *Pten*^*+/+*^ and *Pten*^*398A/398A*^ MEFs cells exposed or not (CTR) to 10 Gy of ionizing radiation, fixed at the indicated times post-IR, and immunostained for PTEN. **B** Quantification of membrane-associated PTEN fluorescent signal, relative to the total membrane area of the cell (*t* test, ****p < 0.0001). **C** Immunoblot analysis of membrane-associated PTEN in *Pten*^*+/+*^ and *Pten*^*398A/398A*^ MEFs cells, exposed or not (CTR) to 10 Gy of ionizing radiation. **D** Quantification of immunoblot data shown in panel (**G**). **E** Model of the effect of phosphorylation of PTEN by ATM on PTEN subcellular localization, PI3K pathway activity, and CDK2/CycE-dependent cell cycle checkpoint regulation, in response to DNA damage.

Given that mutation of the ATM phosphorylation site on PTEN does not appear to intrinsically affect PTEN lipid phosphatase activity under physiological conditions (Supplementary Fig. 3B), we next sought to explain why phosphorylation of AKT was enhanced following DNA damage in cells expressing PTEN-398A. Since PTEN functions at the plasma membrane to dephosphorylate phosphatidyl-inositol 3, 4, 5 phosphate (PIP_3_), we investigated its cellular localization upon DNA damage. MCF10A cells were synchronized and irradiated as described above, and the subcellular distribution of PTEN was analyzed by immunofluorescence. The PTEN-WT and PTEN-398A proteins were similarly localized at steady state in the nucleus and at the plasma membrane (Fig. 6A, B). Following IR treatment, PTEN-WT accumulated at the plasma membrane (Fig. 6A, B). By contrast, no relocalization of PTEN-398A was detected in these conditions (Fig. 6A, B). We further confirmed these results by cellular fractionation and immunoblot analysis of membrane-associated PTEN (Fig. 6C, D). These results suggest that phosphorylation of PTEN by ATM causes PTEN redistribution at the plasma membrane, which is associated with reduced AKT pathway activation.

## Discussion

Early studies in the PI3K field considered PTEN to be a ubiquitously expressed phosphatase functioning as a negative regulator of the kinase pathway, rather than a dynamic signaling molecule. Subsequent studies challenged this initial belief, revealing that PTEN activity is subject to extensive regulation mainly by post-translational modification. We previously identified how sumoylation of PTEN at position 254 controls its nuclear localization and function. We also observed that sumoylation of PTEN is mutually exclusive with its phosphorylation by ATM at position 398 [27]; however, the physiological implications of this regulation event were unknown. In the present work, we uncovered a novel mechanism whereby the kinase ATM affects the subcellular distribution of PTEN, in turn controlling the activity of the PI3K pathway in the context of the DNA damage checkpoint. It is currently unclear how this regulation is implemented at the molecular level. The phosphorylation of PTEN at position 398 could alter its interaction with proteins that control its localization whithin the cell. Alternatively, the addition of a phosphate group could induce conformational changes to PTEN protein and alter its ability to interact with the lipid membrane as it has been proposed by several structural biochemistry studies [44, 45].

We studied the effect of the *Pten*^*398A*^ mutation on mammary tumors driven by the HER2/neu oncogene in *MMTVNeu* transgenic mice. This model has been shown to recapitulate the gene expression profile of human luminal B breast cancers, and has been used to test clinically relevant therapies and identify potential biomarkers of drug sensitivity and resistance [46]. The acceleration in the tumorigenesis process we observed in *MMTVneu*;*Pten*^*398A/398A*^ mice compared to *Pten*^*+/+*^*;MMTVneu* littermates is characterized by a decrease in apoptosis, and an increase in genome instability, which can be attributed to a defective activation of the DDR checkpoint. Interestingly, previous studies showed that the *MMTVneu* model is particularly susceptible to additional mutations in DDR regulators, as is the case in bi-transgenic mice expressing the HER2 oncogene and an inactivated form of *p53* (WAP-p53-172H) with a tumor latency shortened to 154 days (compared to 205 days of the *MMTVneu*) [47].

By using two different cell systems, we were able to dissect the molecular mechanism responsible for the in vivo phenotype and reveal a novel regulatory function of ATM, one of the main tumor suppressors in cells. p27^Kip1^ negatively regulates the G1–S phase progression by binding to and inhibiting cyclin dependent kinases (CDKs), and low levels of p27^Kip1^ correlate with poor prognosis and survival in many types of cancers [48]. However, in human cancers, genetic alterations of p27^Kip1^ are rare. Rather, p27^Kip1^ is misregulated in tumors by transcriptional and post-transcriptional mechanisms. Based on our results, we suggest a model whereby expression of a PTEN form which is resistant to the kinase activity of ATM leads to an impairment of the ability of p27^Kip1^ to bind the CDK2/CycE complex and properly arrest the progression of the cell cycle following genotoxic stress (Fig. 6E). Our data are in accordance with several studies that have shown how the functional impairment of p27^Kip1^ plays a prominent role in breast cancers. Specifically, mislocalization of p27^Kip1^, which is regulated by phosphorylation events [49], in HER2 positive breast cancer cells confers resistance to anti-HER2 targeted therapy [50].

Cell cycle checkpoints play a pivotal role in the control of genetic stability, and their disruption is closely associated with malignant transformation [51]. DNA repair mechanisms ensure the maintenance of genomic integrity over a cell’s lifespan. DNA damage checkpoints, on the other hand, are responsible for determining the fate of genomically unstable cells by activating cell cycle arrest and, ultimately, apoptosis. Disruption of these molecular processes in cells not only increases their likelyhood of neoplastic transformation, but also has therapeutic implications. DNA damage checkpoints directly affect the sensitivity of cells to damage during chemo- or radiotherapy. Therefore, assessing the integrity of checkpoints in cancerous lesions may assist in the design of better therapeutic strategies. This can include the specific targeting of checkpoint components that are defective in certain cancer cells, in order to enhance the antitumor effect of chemotherapies by restoring the sensitivity to cell-cycle arrest and apoptotic cell death. By unveiling a novel mechanism connecting the DDR, cell cycle checkpoints, and PTEN, a key tumor suppressor that is often disrupted in cancers, our study has therapeutic implications for the clinical development of a number of new anticancer drugs that target various cell cycle regulators.

## Supplementary information

Supplementary material
