# Supplementary material for: The PTEN and ATM axis controls the G1/S cell cycle checkpoint and tumorigenesis in HER2-positive breast cancer
Source: Cell Death Differ. 2021 May 31;28(11):3036–51. doi: 10.1038/s41418-021-00799-8 (PMC8564521; doi:10.1038/s41418-021-00799-8)
Supplement: Supplementary file 1 — Supplementary material [file 41418_2021_799_MOESM1_ESM.pdf]

Supplementary figure 1: Genetic engineering of *MMTVneu* and *Pten$^{398A/398A}$;MMTVneu* mammary tumor mouse models. (A) Schematic for the strategy to engineer the *Pten^{398A}* allele.. (B) Breeding scheme to generate *Pten$^{398A/398A}$;MMTVneu* mice

Supplementary figure 2: H&E, HER2 and Ki-67 immunostaining of tumor tissue from *MMTVneu* and *Pten$^{398A/398A}$;MMTVneu* mammary tumors. (A) Representative images of H&E-stained histological sections from mammary tumors from *MMTVneu* and *Pten$^{398A/398A}$;MMTVneu* mice. (B) Representative images of HER2 IHC analysis of mammary tumor tissue from *MMTVneu* and *Pten$^{398A/398A}$;MMTVneu* mice. (C) Representative immunostaining images and (D) quantification of Ki-67 IHC analysis of mammary tumor tissue from *MMTVneu* and *Pten$^{398A/398A}$;MMTVneu* mice.

Supplementary figure 3: PTEN and PTEN$^{398A/398A}$ protein expression levels. (A) Immunoblot analysis of total PTEN and PTEN 398A protein in MEFs isolated from *Pten* and *Pten$^{398A/398A}$* mice. (B) Immunoblot analysis of total PTEN and PTEN 398A protein in MEFs isolated from *Pten$^{+/+}$* *and Pten$^{398A/398A}$* mice (lanes 1 and 2) and MCF10A *PTEN null* cells reconstituted with FLAG-PTEN and FLAG-PTEN 398A (lanes 3 and 4, respectively).

Supplementary figure 4: Defective repair of DNA damage in cells expressing PTEN 398A. (A) Representative images of MCF10A cells expressing FLAG-PTEN or FLAG-PTEN 398A exposed or not (CTR) to 5 Gy of ionizing radiation, fixed at the

indicated times post-IR, and immunostained for γH2AX, and 53BP1. DAPI was used to stain DNA. (B) Quantification of the number of γH2AX nuclear foci in MCF10A cells expressing FLAG-PTEN or FLAG-PTEN 398A. Mean and standard error of the mean are indicated for each transfection. Nuclear foci in at least 100 nuclei were counted. (t-test, ***denotes $p < 0.001$). (C) Quantification of the number of 53BP1 nuclear foci in MCF10A cells expressing FLAG-PTEN or FLAG-PTEN 398A. Mean and standard error of the mean are indicated for each transfection. Nuclear foci in at least 100 nuclei were counted. ****denotes $p < 0.0001$. (D and E) Summary of I-*Sce*I–based Homologous Recombination and Non-Homologous End Joining assays. HR and NHEJ assays were established as described previously (48).

Supplementary figure 5: MCF10A cells expressing PTEN 398A are more resistant to genotoxic stress. (A and B) Heatmap, representing signal intensity, from antibody microarray analysis of lysates from MCF10A cells expressing FLAG-PTEN (A) or FLAG-PTEN 398A (B). Each row indicates a specific antibody tested in the antibody array experiment, and each column indicates an individual condition: ctr, control; 30’, 30 minutes after irradiation (5Gy); 4h, 4 hours after irradiation and 24h, 24 hours after irradiation. 398AThe intensity increased from yellow color to red color. (C to E) Survival of MCF10A cells expressing FLAG-PTEN or FLAG-PTEN 398A, over 6 days, in untreated cells (C), or following exposure to 5 Gy of ionizing radiation (D), 1uM and 5um Cisplatin (E). (F) Number of 53BP1 and γH2AX foci in MCF10A cells expressing FLAG-PTEN or FLAG-PTEN 398A and treated with 20 μM z-VAD-fmk or vehicle (DMSO), 24 hours after 10 Gy irradiation or without irradiation. z-VAD-fmk was added 2 hours prior

to irradiation. All data are expressed as mean ± SD. Experiments in triplicate. t-test, * $p < 0.05$, ***<0.001 compared with corresponding controls

Supplementary figure 6: Transcriptional profile of PTEN 398A MCF10A cells following genotoxic stress. (A) GSEA plots of most significantly upregulated gene sets in MCF10A cells expressing FLAG-PTEN 398A, compared to MCF10A cells expressing FLAG-PTEN, following genotoxic stress (5 Gy IR). Genes are ranked from the most up-regulated (left end) to the most down-regulated (right end). The “barcode” indicates the position of the genes from the dataset in respect to the gene set of the biological pathway being interrogates. The normalized enrichment score (NES) is inferred from permutations of the gene set and the false discovery rate (FDR). P values are indicated.

Supplementary figure 7: Cell cycle profiling of MCF10A cells. (A) Schematic of the experimental approach for the cell cycle analysis. (B) Cell cycle profile of MCF10A cells expressing FLAG-PTEN or FLAG-PTEN 398A. x axis indicates the time after release from thymidine block.

A

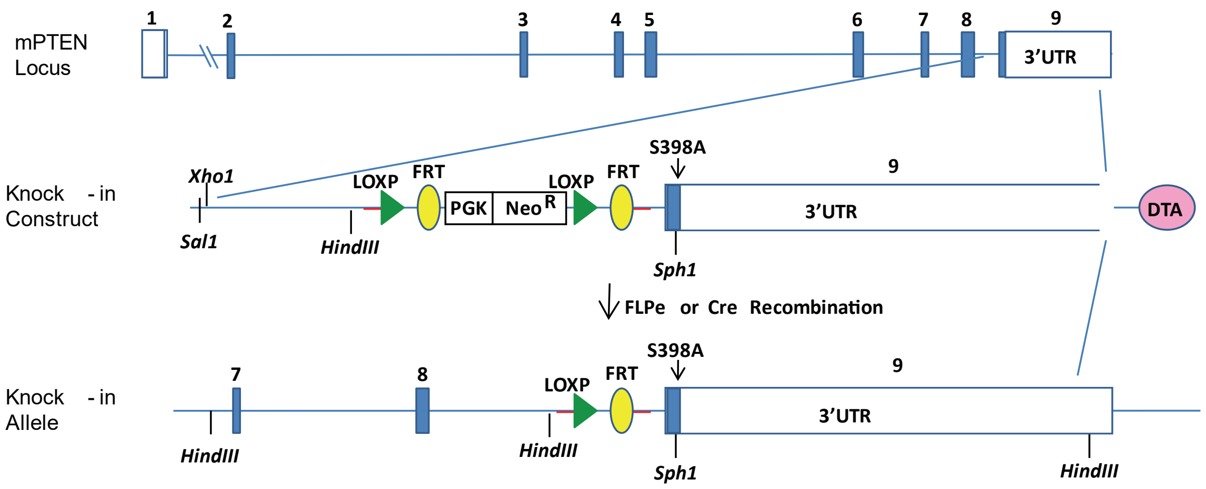

B

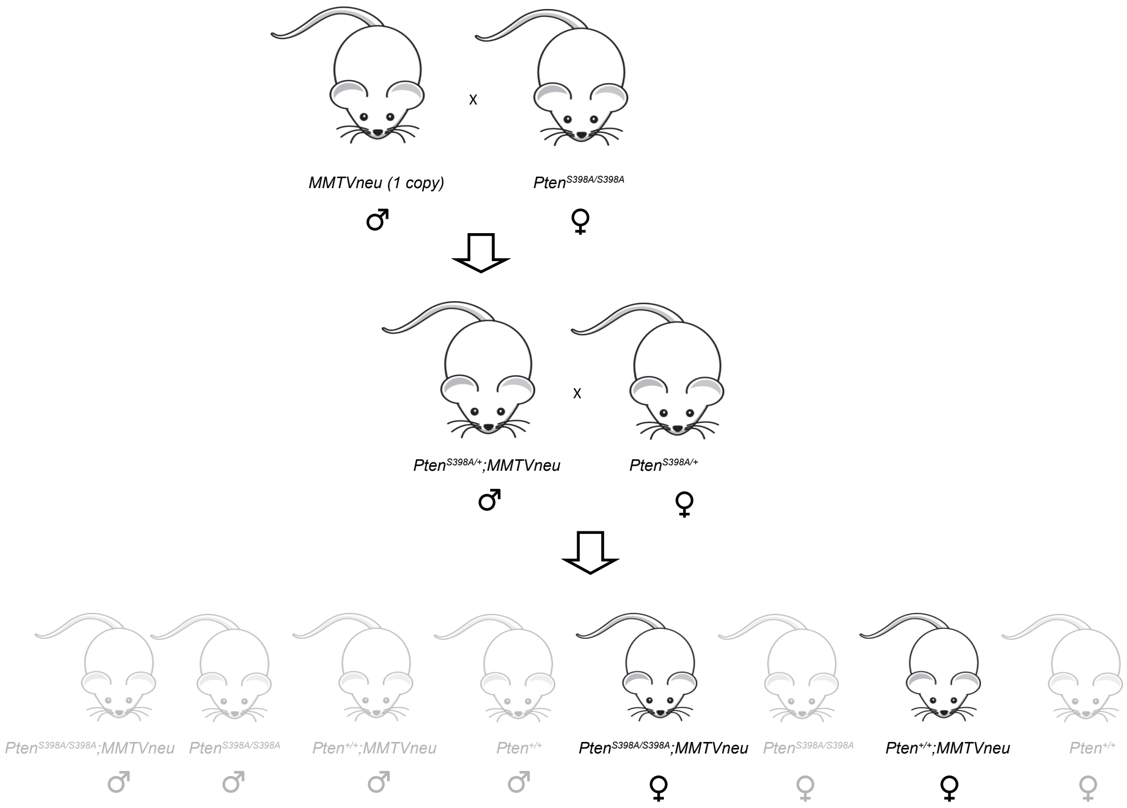

Supplementary figure 1

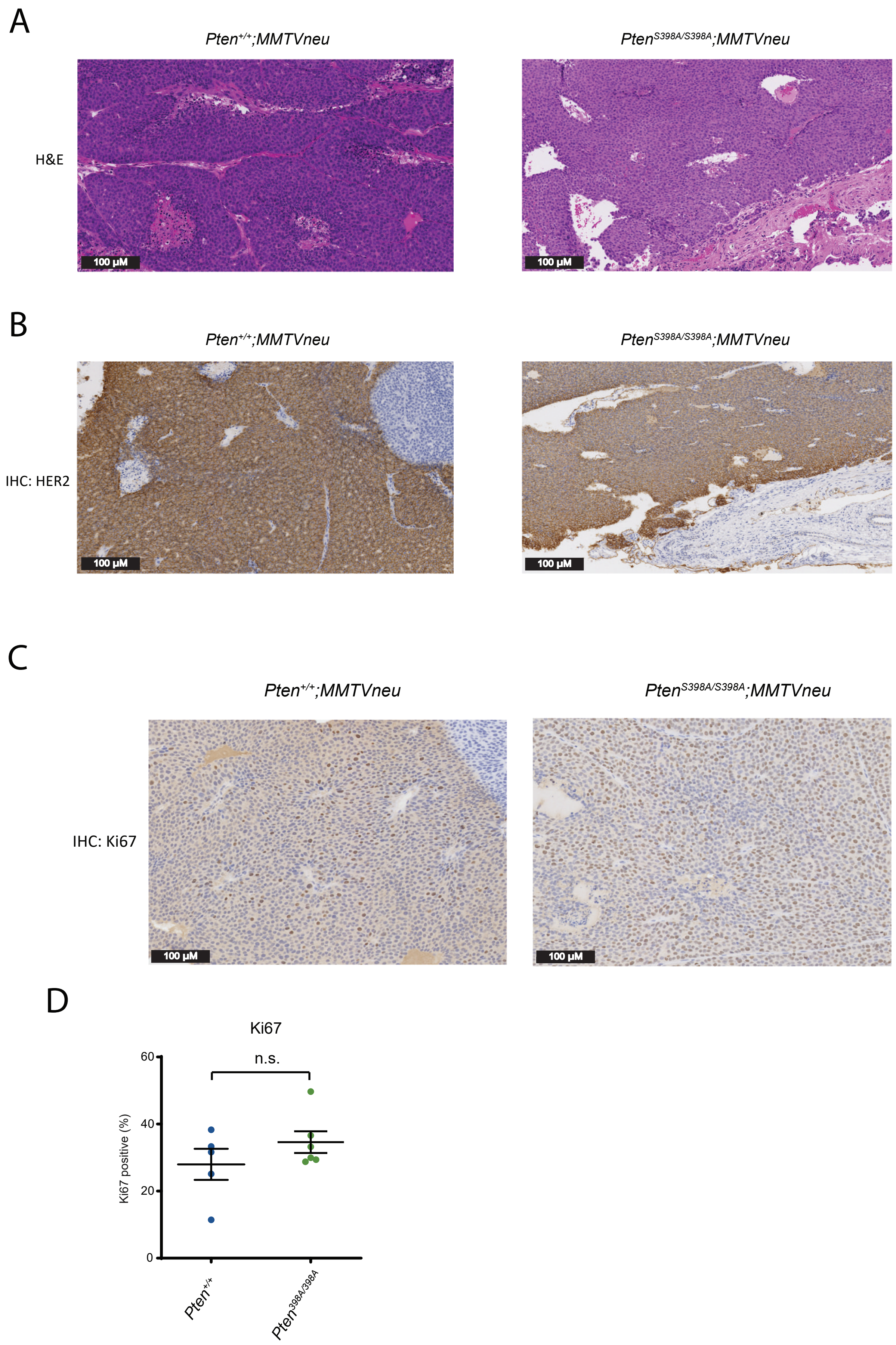

Supplementary figure 2

A

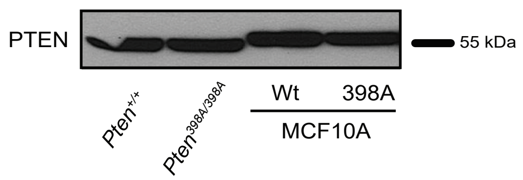

B

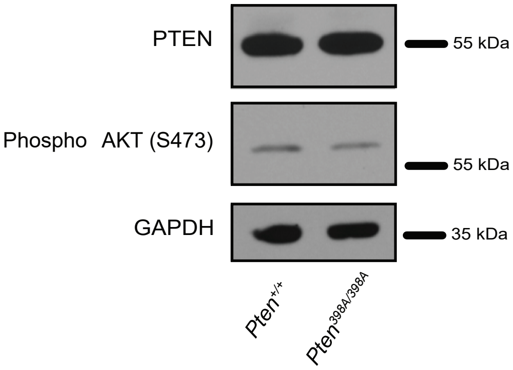

Supplementary Figure 3

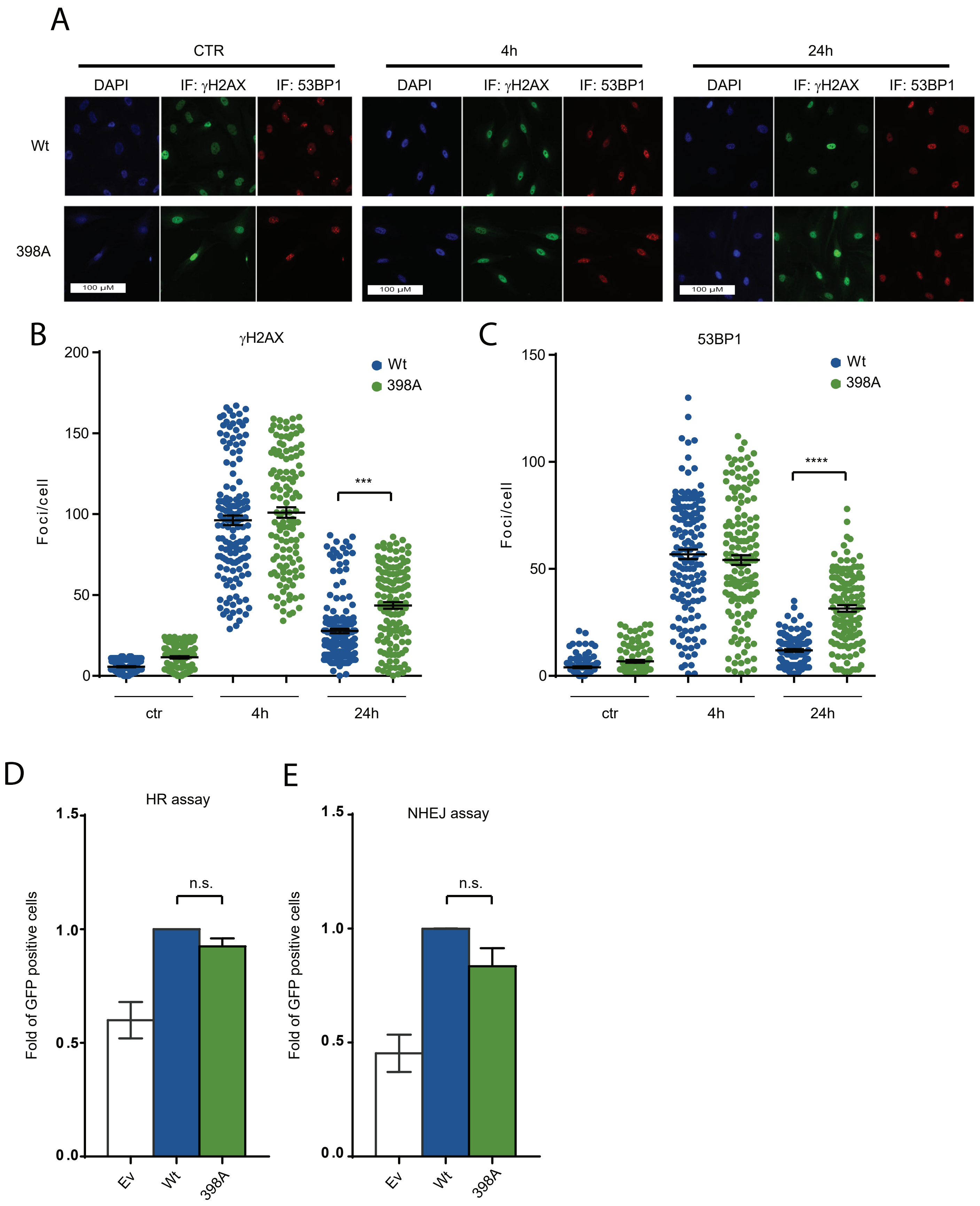

Supplementary Figure 4

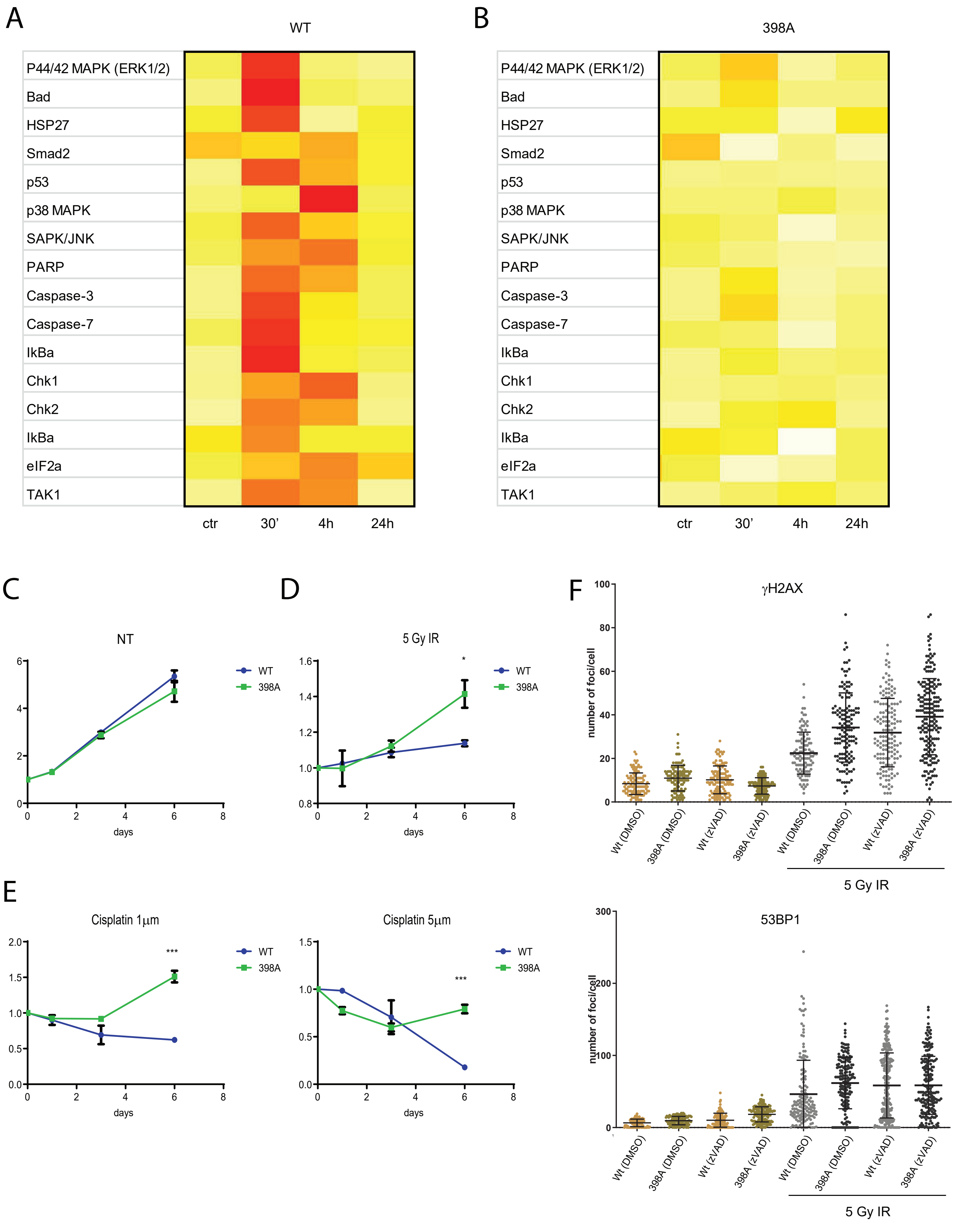

Supplementary Figure 5

A

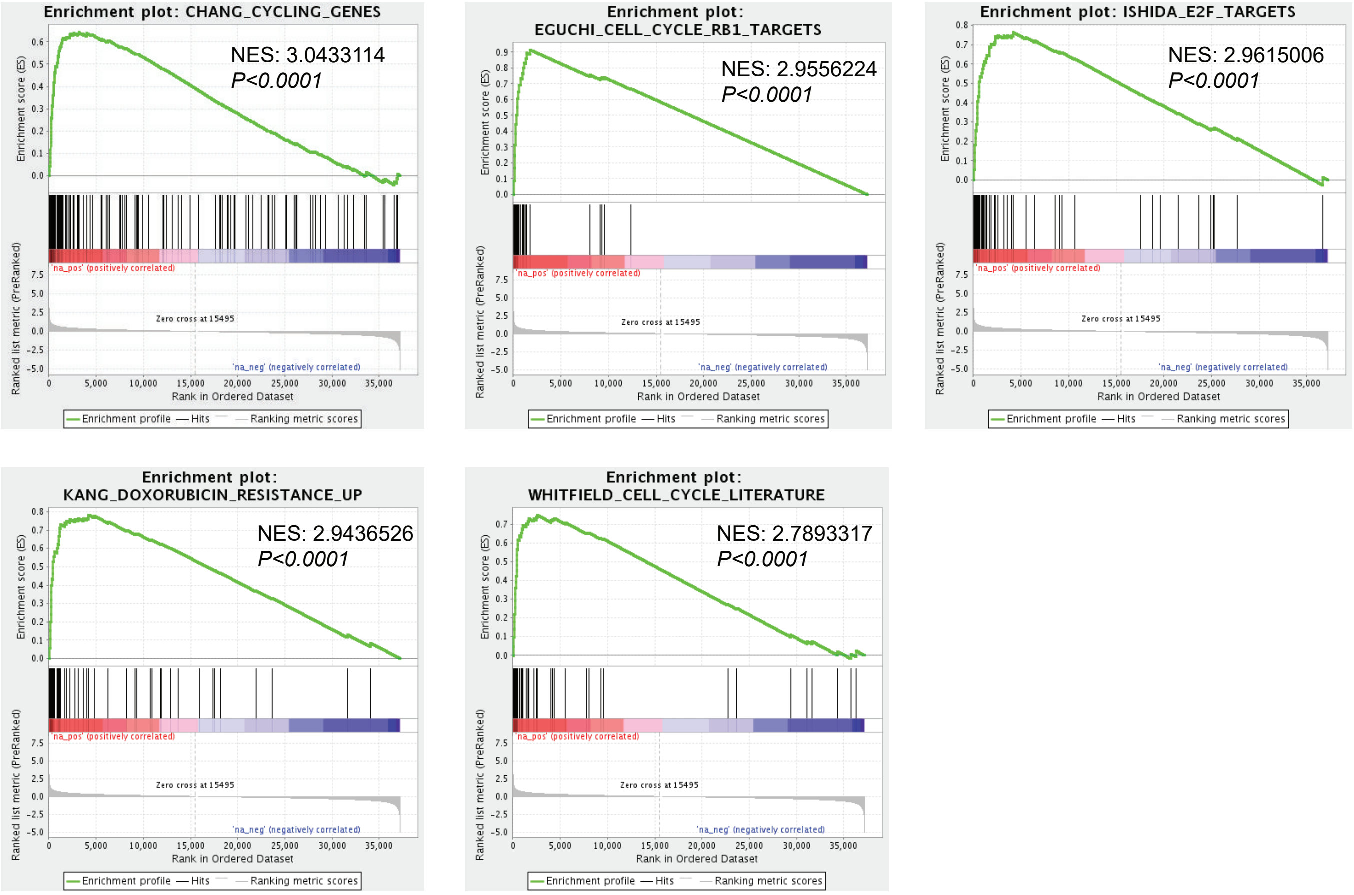

Supplementary Figure 6

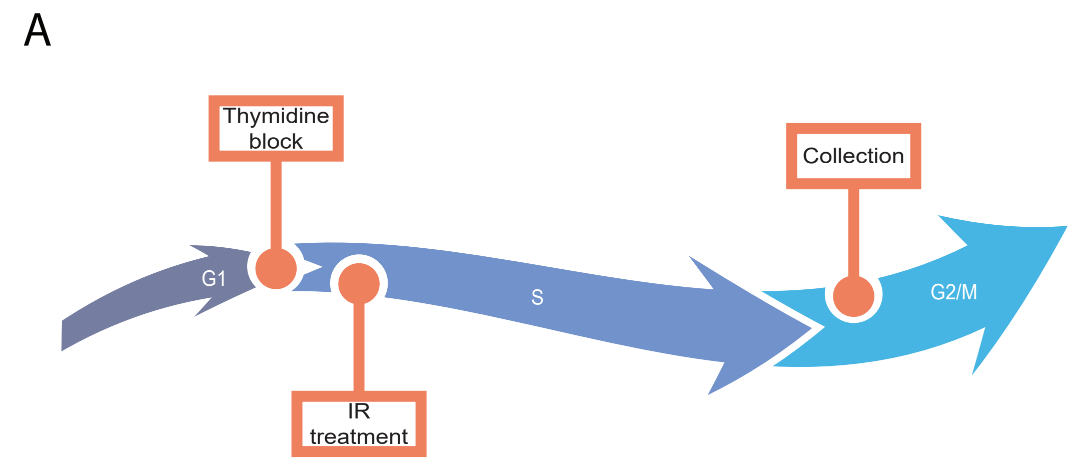

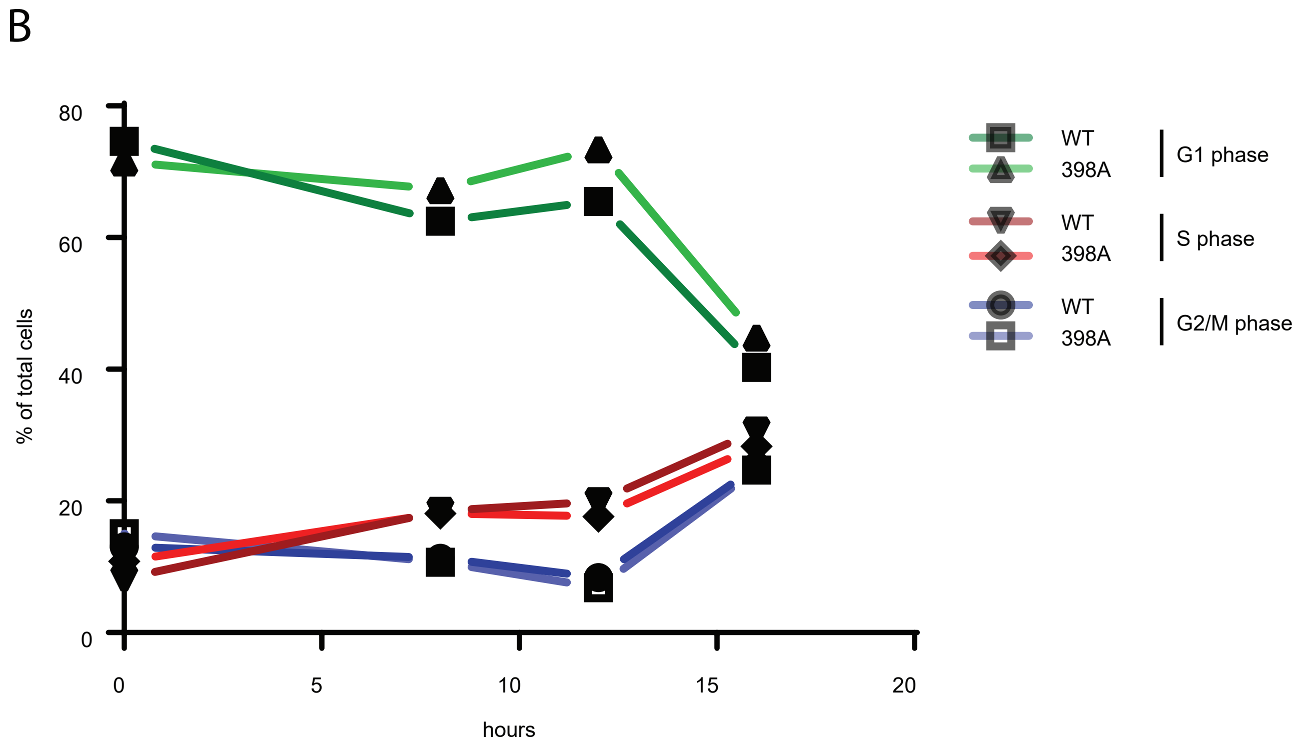

Supplementary Figure 7
